# Terahertz absorption characteristics of ammonium salt solution based on self-sampling microfluidic chip

**DOI:** 10.1038/s41598-022-11858-6

**Published:** 2022-05-17

**Authors:** Qinghao Meng, Siyu Qian, Jing Ding, Qingjun Li, Xinyuan Zhao, Bo Su, Cunlin Zhang

**Affiliations:** 1 Key Laboratory of Terahertz Optoelectronics, Ministry of Education, Beijing, 100048 China; 2 Beijing Key Laboratory for Terahertz Spectroscopy and Imaging, Beijing, 100048 China; 3 Beijing Advanced Innovation Centre for Imaging Theory and Technology, Beijing, 100048 China; 4 Department of Physics, Capital Normal University, Beijing, 100048 China

**Keywords:** Lab-on-a-chip, Microfluidics, Spectrophotometry, Chemical physics

## Abstract

With the continuous development of terahertz (THz) detection technology, the use of terahertz spectroscopy to study chemical samples has become one of the indispensable tools in the field of biochemistry. While most biomolecules biological activity can only be expressed in aqueous solutions, water as a polar molecule has strong absorption properties for terahertz waves, making it difficult to use terahertz technology to study the activity of biological samples in aqueous solutions. In this study, a sandwich-type terahertz microfluidic chip with high terahertz wave transmission was designed and combined with a terahertz time domain spectroscopy (THz-TDS) system to test the terahertz spectra of distilled water, 0.9 mol/L NH_4_Cl, (NH_4_)_2_SO_4_, (NH_4_)_2_CO_3_ and CH_3_COONH_4_ solutions, respectively, and to investigate the effect of the electric field action time on the hydrogen bond in the solution under the action of an external electric field. The experimental results show that the terahertz spectra of different ammonium solutions at the same concentration differ significantly, indicating that the ion hydration process affects the intermolecular hydrogen bonding in water, while the applied electric field also affects the hydrogen bonding in water, resulting in a change in the terahertz waves water absorption.

## Introduction

Terahertz (THz) wave are electromagnetic waves with a frequency range of 0.1–10 THz and a wavelength range of 0.03–3 mm^1–4^. THz waves are the intersection of macroscopic electronics and microscopic photonics because their long wave band coincides with millimeter wave (submillimeter wave) and their development is primarily dependent on electronic technology; their short wave band coincides with infrared (far infrared) and their development is primarily dependent on optoelectronic technology. Because many weak interactions between molecules, rotational and vibrational jumps of dipoles, and low-frequency vibrational absorption of lattices in crystals fall within the THz band, and THz spectroscopy is extremely sensitive to detecting small differences in the structure of substances. Therefore, THz spectroscopy is widely used in the analysis and research of chemical molecules and other substances. Smolyanskaya et al.^5^ measured free/bound water in diabetic animals’ skin, kidney, and cornea. It has been discovered that the THz-TDS method can reflect the dynamic response of water in biological medium to changes in osmotic pressure. The basic parameters are related to the amount of hydrogen in the sample and change significantly as the water state changes. Joti Y et al.^6^ used THz-TDS system to study and analyze the offsetting phenomenon between autocorrelation and cross-correlation contributions of protein and water in dynamics. Wang et al.^7^ experimentally studied the THz spectra of DNA nuclease crystals using a THz-TDS system, and analyzed the vibration spectra of solid-state DNA nuclease by generalized energy fragmentation method (PBC-GEBF) under periodic boundary conditions, and assigned the corresponding vibration modes to the main peaks in the experimental spectrum. The harmonic vibration frequency results show that all of the vibration modes belong to collective vibration modes, which involve complex mixtures of intermolecular and intramolecular displacements, in the 0.5–9 THz range.

In experiments using THz-TDS system to explore the properties of biomacromolecules, the strong interaction between the low-frequency intermolecular vibrations involved in hydrogen bonds in water molecules and THz waves causes most of the THz to be absorbed by water, making it particularly difficult to study the target sample in liquid environment. Microfluidic technology has emerged as a critical means of addressing the aforementioned problems in the context of increasingly intelligent, modern, and miniaturized MEMS technology. Yang et al.^8^ designed a fast and label-free cell viability measurement method. The method uses THz spectroscopy, microfluidic technology, and a new optical scavenger (OCA) that replaces highly absorbed water molecules around living cells with a relatively small amount of OCA, while reducing background signal interference. Mathias et al.^9^ designed a temperature and pressure controllable microfluidic chip to contain A549 lung cancer cells inside the chip and concluded that they could be exposed to 1MPa standing wave acoustic pressure for 1 hour without affecting cell viability. Lin et al.^10^ developed a microfluidic chip with an integrated microheater and a luminescent temperature sensor for rapid spatial melting curve analysis, which they applied to screening of breast cancer gene fragments.

In this study, a THz microfluidics chip with automatic sample entry and exit function is designed and implemented for the transmission THz-TDS system. The detection region is made of COC material, which is colorless and transparent, has no noticeable absorption peaks in the THz frequency range, and is highly permeable to THz waves, with a transmission rate of more than 90% (As shown in Figure S1) for a 2 mm thick COC material^11^. To achieve automatic injection, a valveless micropump was designed on the chip surface. Ammonium salt has strong bactericidal, mildew and moth proof effects. It is a common salt in daily life. At the same time, its hydration characteristics also have good research prospects. The chip was used to measure distilled water, NH_4_Cl, (NH_4_)_2_SO_4_, (NH_4_)_2_CO_3_ and CH_3_COONH_4_ solutions, respectively. Transmission spectra were obtained in the 0.1–0.6 THz frequency range. It was discovered that the four kinds of anions in the solution all promoted the formation of hydrogen bonds between water molecules. Then, in order to explore the effect of the applied electric field on the solution, the chip was used to study how the THz transmission intensity of four ammonium solutions changed under the applied electric field at different times. The results show that the THz transmission intensity of the various ammonium solutions tends to increase as the resting time in the electric field increases, indicating that the applied electric field breaks the hydrogen bonds in the ammonium solutions and that the breaking effect increases with time.

## Methods

### THz time domain spectroscopy system

**Figure 1:**
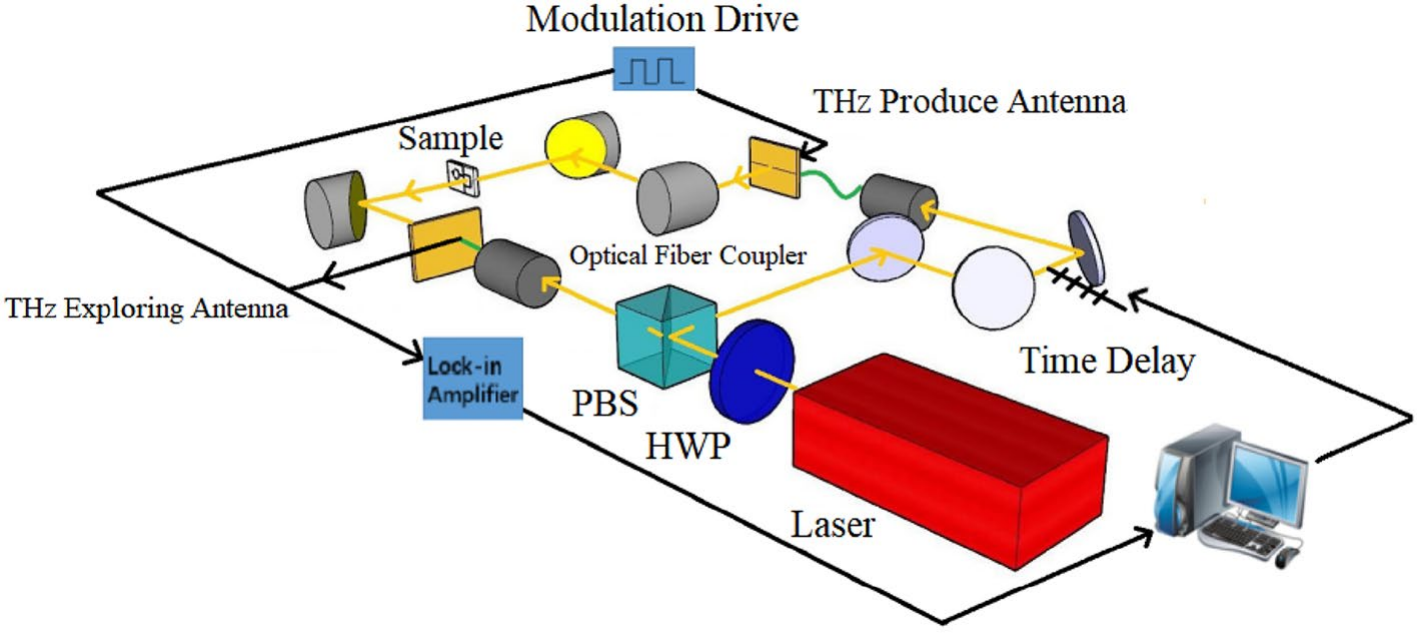
THz time domain spectroscopy system.

Figure 1 depicts the THz-TDS system, which consists of a femtosecond laser, a THz radiation generation device, a corresponding detection device, and a time-delay control system. The research uses a self-mode-locked fiber femtosecond laser developed and customized by Peking University (central wavelength is 1550 nm, pulse width 75 fs, pulse repetition rate 100 MHz, and output power 130 mW). A polarization splitting prism was used to split the impulse laser into two beams. To generate THz waves, one beam was coupled into an optical fiber photoconductive antenna (BATOP company bPCA-100-05-10-1550-c-f) via a mechanical translation platform, and the other was coupled into another optical fiber photoconductive antenna (BATOP company bPCA-180-05-10-1550-c-f) to detect the THz waves. The microfluidic chip is fixed between the off-axis parabolic mirrors, and the THz wave generated by the THz transmitting antenna passes via the chip filled with liquid sample, carrying sample information, which is received by the detecting antenna and input to the phase-locked amplifier for amplification. Finally, the computer collects and processes the data.

### External high-voltage electric field test equipment

**Figure 2:**
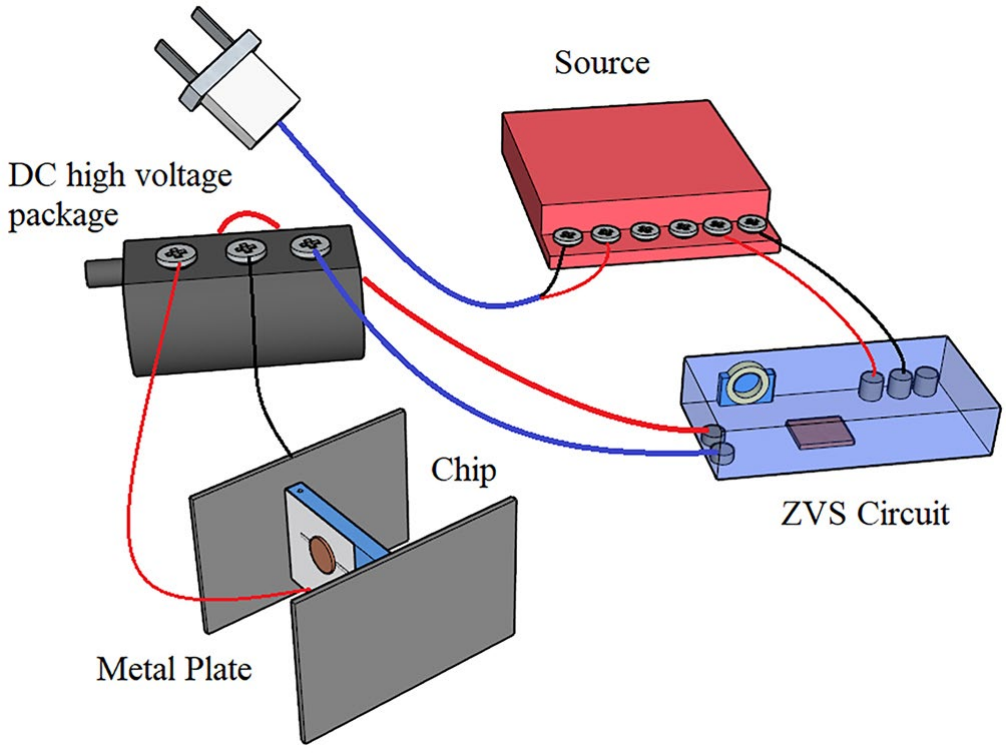
Schematic diagram of applied high-voltage electric field experimental device.

Figure 2 depicts the external high-voltage electric field test equipment. The experimental apparatus consists of a power supply, a zero voltage switching (ZVS) circuit encapsulated in a PMMA box, and a DC high-voltage package with a 10000 V output voltage. The power supply is wired to the ZVS circuit, the other end of the ZVS circuit is wired to the high-voltage package, and the output of the high-voltage package is wired to two directly opposite metal plates. The two metal plates are 8 cm × 15 cm in size, and the microfluidic chip is placed in the center of the two metal plates. The distance between the two metal plates is 4 cm, and the applied electric field has a strength of 2500 V/cm.

### Preparation of microfluidic chips

**Figure 3:**
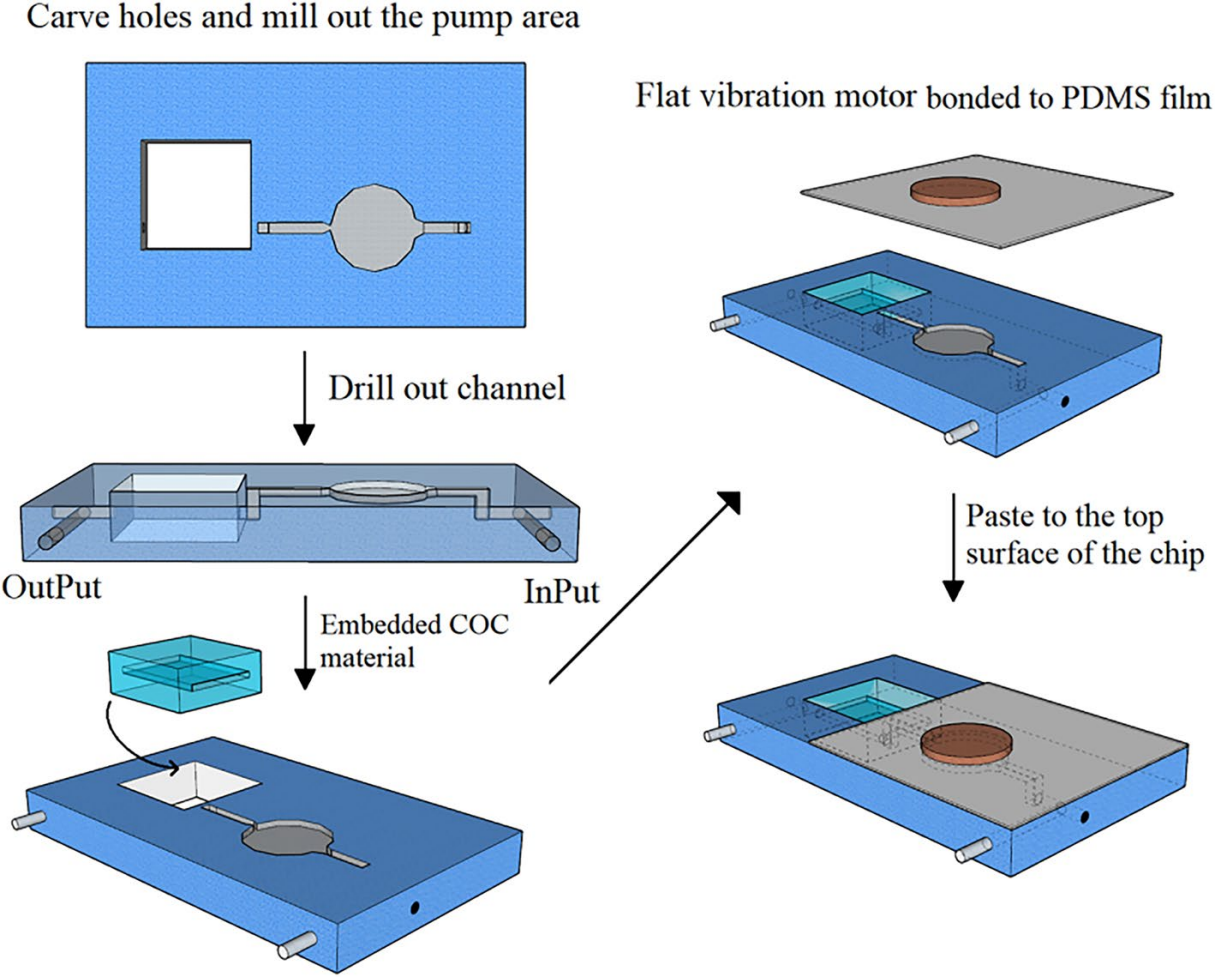
Preparation flow of embedded COC THz microfluidic chip with self-sampling capability.

**Figure 4:**
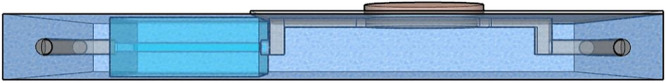
Side view of the microfluidic chip.

The embedded COC THz microfluidic chip’s preparation method is as follows. A laser engraving machine is used to cut a piece of PMMA with a thickness of 2 mm into a rectangular parallelepiped with a length of 20 mm and a width of 12 mm, and a 5 mm × 5 mm × 2 mm rectangular hole is cut in the area where the COC material is embedded in the PMMA sheet. A 0.3 mm deep pump cavity, a tapered micropump diffuser/spout, and a liquid channel were milled into the surface of PMMA using a milling cutter. The milling depth of the left and right sides of the structure is increased to 1 mm to ensure the tightness of the chip and the fluidity of the liquid. To connect the left and right surfaces of the PMMA, a special drill bit with a diameter of 0.5mm is used, and the drill bit is used again to drill two channels with a length of 4 mm on the left and right sides of the upper side of the PMMA, which are perpendicular and connected to the two channels drilled on the left and right sides. Rubber putty is used to block the channels drilled on the left and right sides of PMMA, and the inner L-shaped channel on the upper left side of PMMA is used as the liquid inlet channel and the inner L-shaped channel on the upper right side of PMMA is used as the liquid outlet channel. A 5 mm long, 3.5 mm wide and 2 mm high COC material is cut out with a cutter, and a 5 mm long, 3.5 mm wide, and 0.3 mm high channel is milled out in the material side’s center area to serve as the liquid sample detection area. The prepared COC material was coated with 502 glue on one side and inserted into the engraving hole on the PMMA chip. As the pump film, a layer of PDMS film is pasted on the surface of PMMA, and a flat vibration motor is pasted on it as the micro pump’s driver. Figure 3 depicts the chip preparation process, while Fig. 4 depicts the chip’s side view.

### Experimental procedure

NH_4_Cl, (NH_4_)_2_SO_4_, (NH_4_)_2_CO_3_, CH_3_COONH_4_ solutions with a molar concentration of 0.9 mol/L are chosen to test the feasibility of the microfluidic chip for measuring liquid samples, the concentration is the maximum at which saturation does not occur. These electrolyte solutions all have the same cation, NH_4_^+^. A flat vibrating motor drives the valveless micropump designed on the chip surface. It has a working voltage of 1.5 V, a frequency of 25 Hz, and a maximum liquid flow rate of 2.2 μL/min.

**Figure 5:**
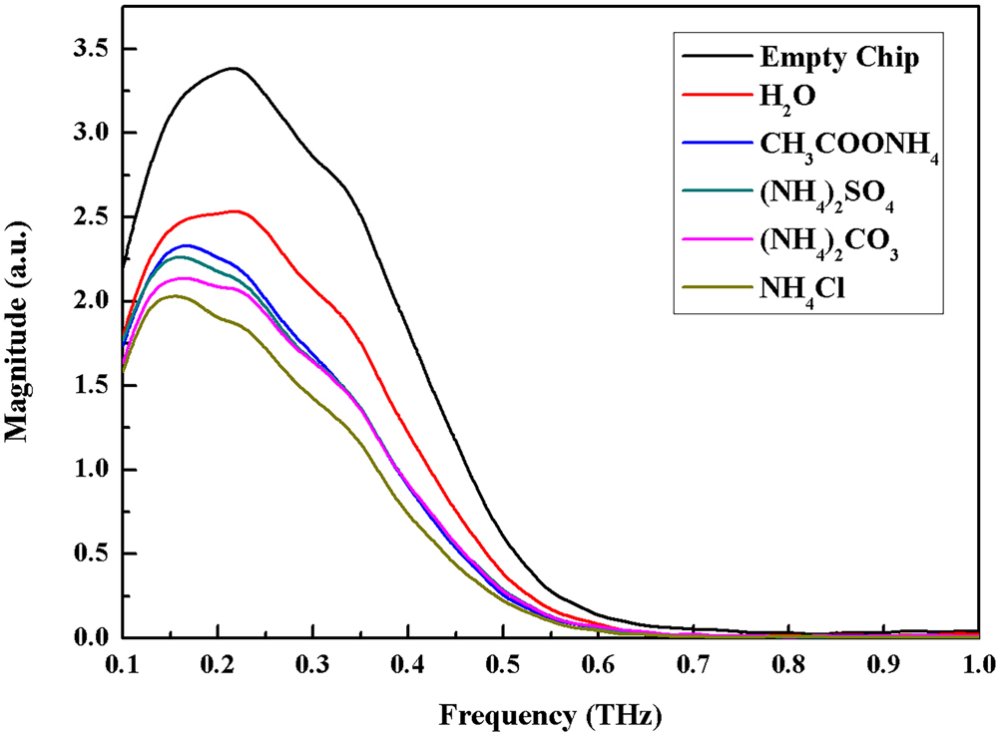
Frequency domain spectra of 0.9 mol/L ammonium salt solution, distilled water, and empty chip.

First, the empty chip without sample injection and the chip with distilled water injection are measured in the self-built THz-TDS system, and the frequency domain spectrum is obtained using the Fourier transform. The prepared ammonium salt solution was then drained to the microfluidic chip in turn by a valveless micropump, and the frequency domain spectrum data was collected. Figure 5 shows the experimental results after data processing.

The spectral intensity of each ammonium salt is found to be lower than that of distilled water. The THz transmission spectra of the four ammonium salts are in the following order: CH_3_COONH_4_ > (NH_4_) _2_SO_4_ > (NH_4_) _2_CO_3_ > NH_4_Cl.

**Figure 6:**
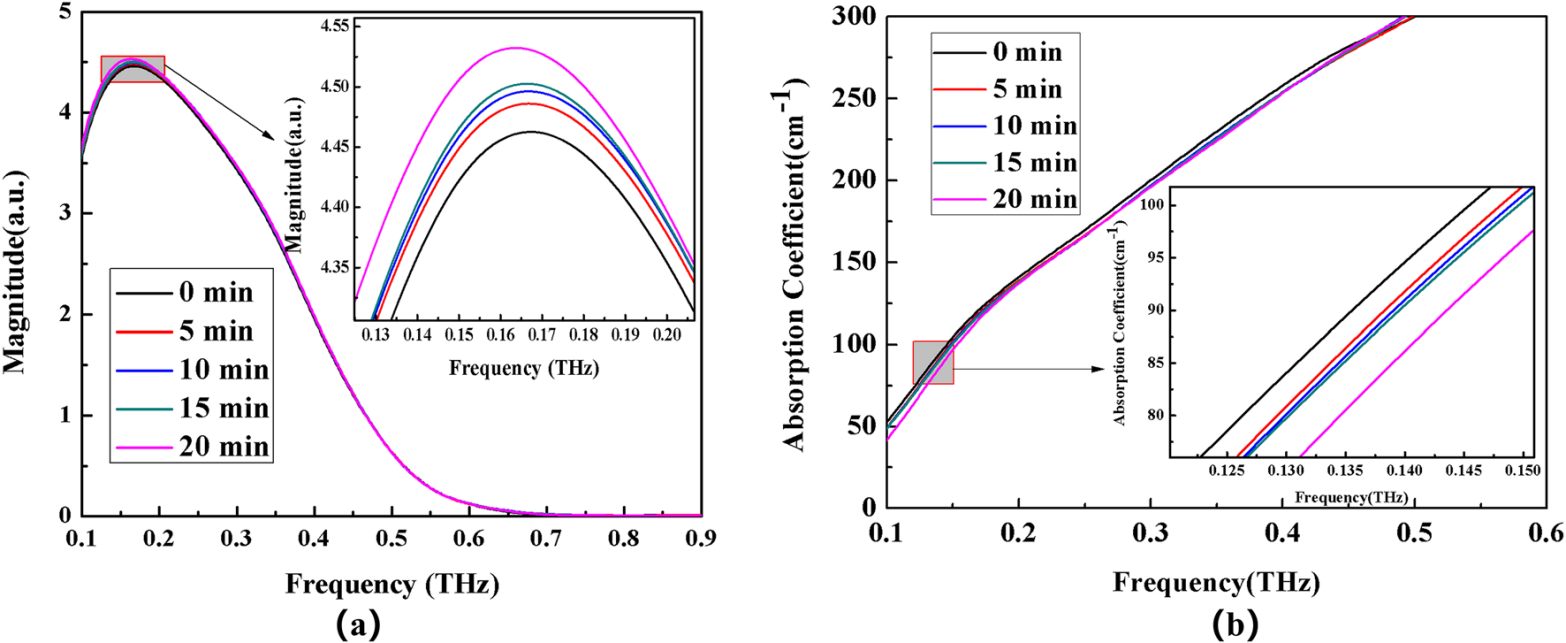
Frequency domain spectrum (**a**) and absorption coefficient spectrum (**b**) of CH_3_COONH_4_ solution with different time electric field applied externally.

**Figure 7:**
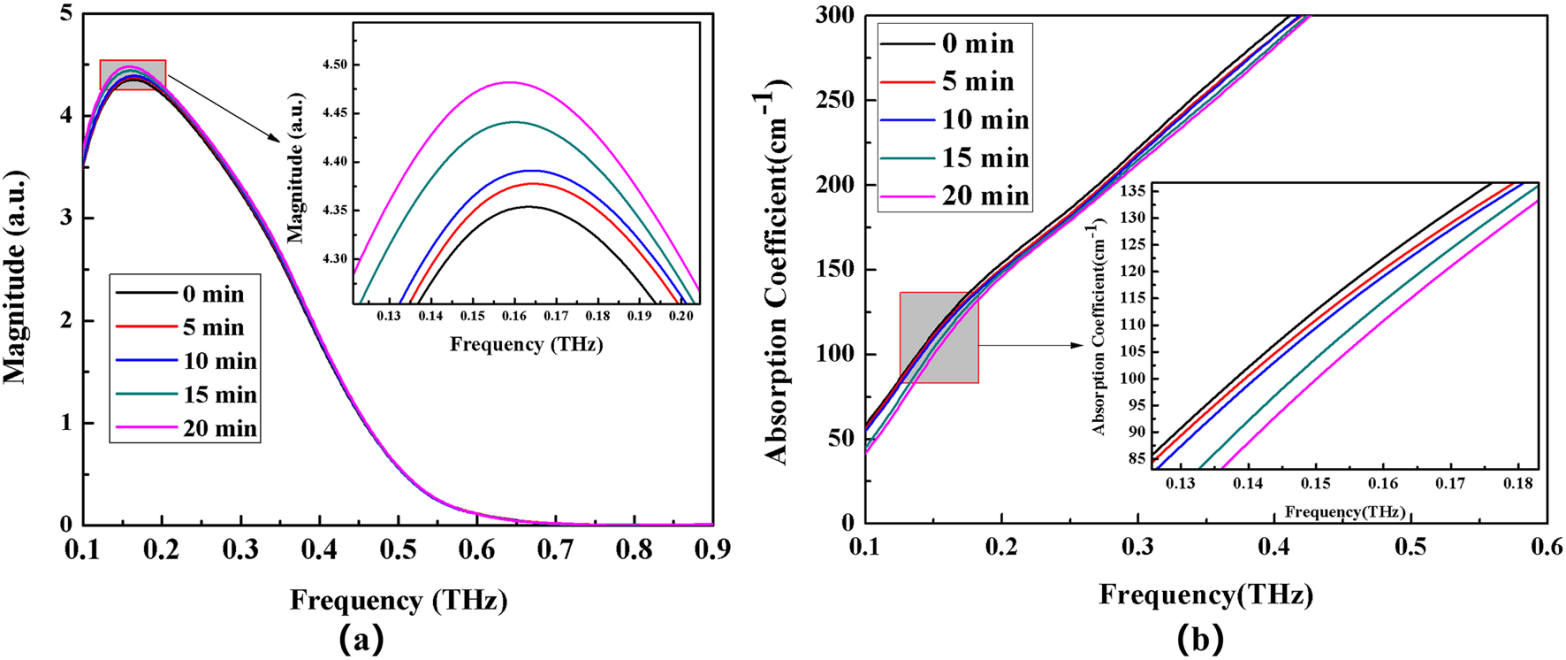
Frequency domain spectrum (**a**) and absorption coefficient spectrum (**b**) of (NH_4_)_2_SO_4_ solution with different time electric field applied externally.

**Figure 8:**
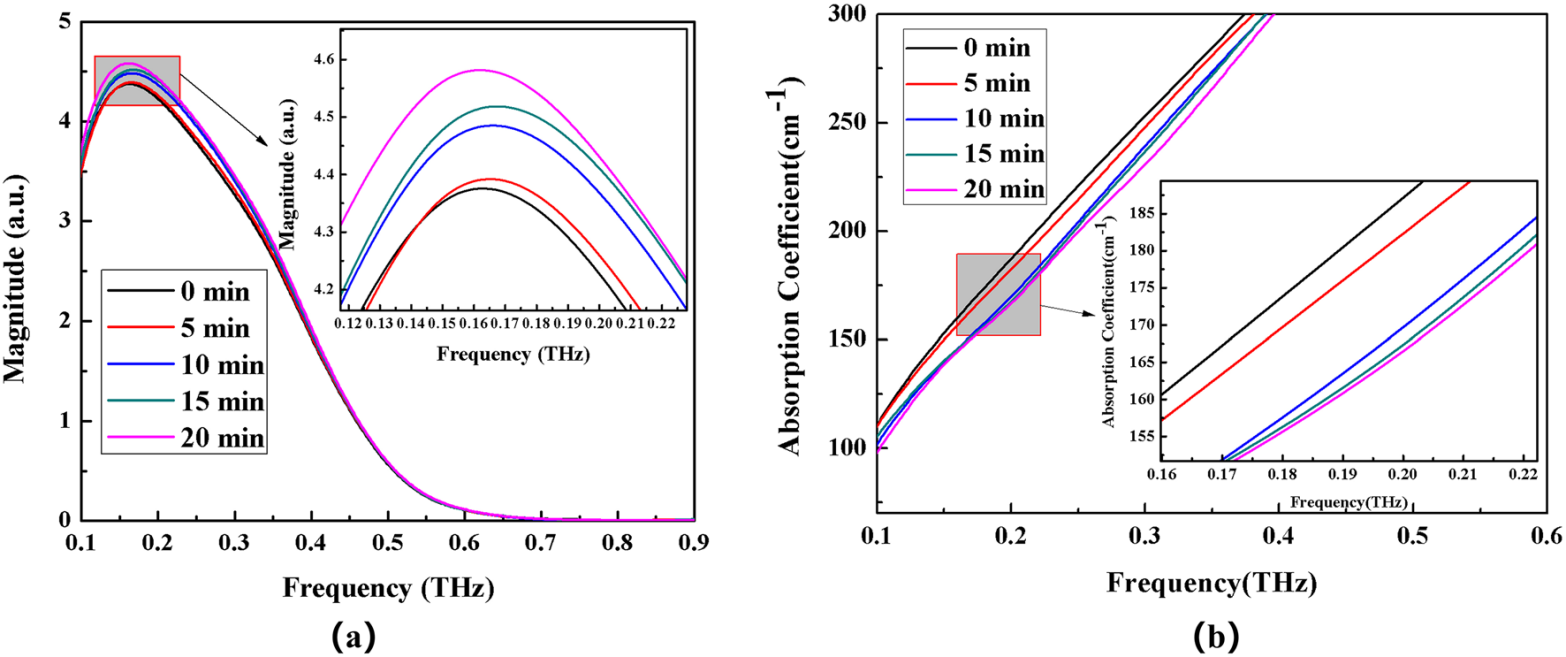
Frequency domain spectrum (**a**) and absorption coefficient spectrum (**b**) of (NH_4_)_2_CO_3_ solution with different time electric field applied externally.

**Figure 9:**
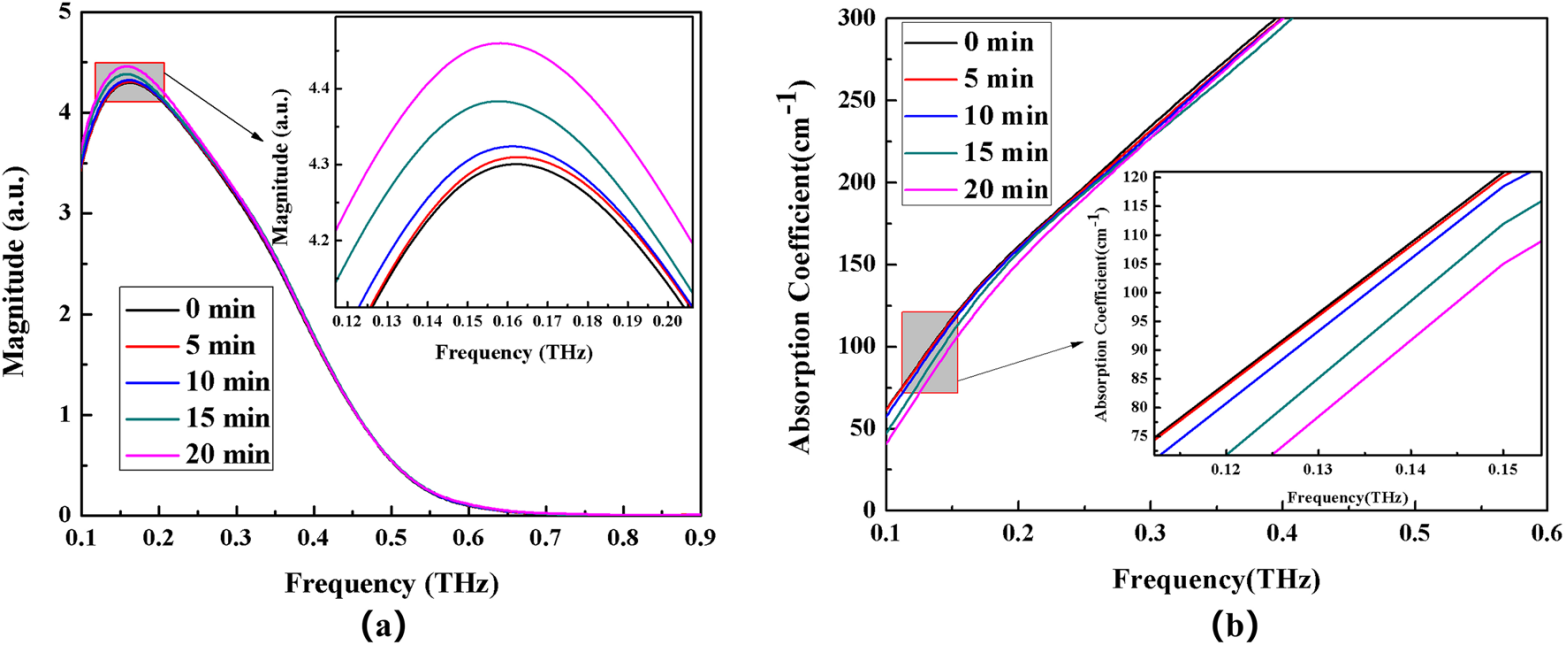
Frequency domain spectrum (**a**) and absorption coefficient spectrum (**b**) of NH_4_Cl solution with different time electric field applied externally.

In the presence of an applied electric field, the solute molecules of the electrolyte solution are reoriented, which in turn affects the hydrogen bonds in the solution. To investigate this phenomenon, four types of 0.9 mol/L ammonium salt solutions were reconfigured and then placed in an electric field at room temperature for standing treatment. The standing times ranges from 0 to 20 min at 5 min interval. The THz-TDS system was then used to test and measure the system's time domain spectrum. The frequency domain spectrum in the range of 0.1–0.6 THz was obtained after Fourier transform. Finally, the absorption coefficient spectrum was calculated. The experimental results after data processing are shown in Figs. 6, 7, 8, and 9.

The THz transmission intensity of the four ammonium salt solutions gradually increases with the increase of the applied electric field time, as observed by observing the variation of spectrum intensity and absorption coefficient of each ammonium salt (the absorption coefficient gradually decreases). THz transmission intensity and standing time in electric field are related as follows: 20 min > 10 min > 15 min > 5 min > 0 min (absorption coefficient and standing time in electric field are related as follows: 0 min > 5 min >10 min >15 min >20 min).

In addition, we carried out a study of the terahertz transmission properties of ammonium salts under different applied electric field strengths. After many previous experiments, no obvious regularity phenomenon was found in ammonium salt solution. However, after our group's research, it is certain that the applied electric and magnetic field of different strengths both have an effect on the terahertz transmission properties of salt solutions, such as C_6_H_5_Li_3_O_7_ (As shown in Figure S1 and Figure S2).

## Results

### Effect of ion hydration on THz transmission characteristics of ammonium salt solution

**Figure 10:**
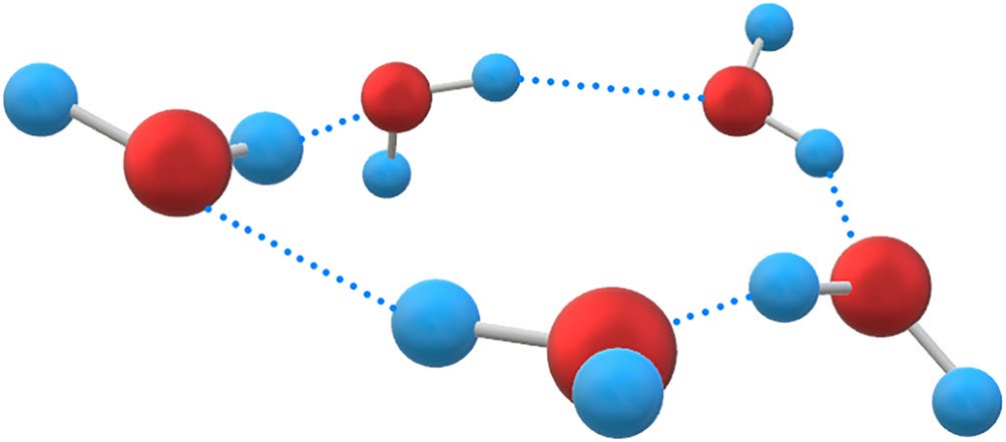
Schematic diagram of the five-ring water structure.

The spectral intensity of distilled water injected into the chip was much lower than that of the empty chip in this study, indicating that water absorbs the vast majority of THz waves, which is primarily due to hydrogen bonding between water molecules; and the difference in spectral intensity between distilled water and the four ammonium salt solutions indicates that ion hydration affects hydrogen bonds in water, and then change the transmission of THz wave to solution. Free water is a cluster of water molecules that are polymerized by hydrogen bonding. Water has a coordination number of 4 in the range of 4–200 °C, according to Liu shiyin^12^, indicating that most of the water cluster is in the form of five-ring water, as illustrated in Fig. 10. However, obtaining the structure of water in a strict sense is more difficult. This is because, while the hydrogen bonding interaction in water is relatively strong, its three-dimensional structure is dynamic, and temperature, electric field, magnetic field, and all kinds of ion hydration can cause fracture and transformation of hydrogen bonding between water molecules.

The hydrogen bonds interaction between water molecules or clusters falls under the category of complex multi-body interaction, which is affected by many factors. In this study, when ammonium salts are dissolved in water to form electrolyte solution, the electric field formed near the ions makes some solvent water molecules aligned around the ions to form hydrated ions, and the microstructure of water in the ion hydration layer changes, which is the influence of ions in the solution on the water structure.

Kitadai et al.^13^ used ATR-IR spectra to explore the effects of different cation and anion ions in the OH stretching band (2600–3800cm^−1^) on the hydrogen bond between water molecules in the hydration layer. The OH stretching band of water is sensitive to the interaction between ions, hydrated water molecules and the hydrogen bond between water molecules. The ions that move the OH stretching band to a lower frequency are usually interpreted as enhancing the hydrogen bond between water molecules in the hydration layer, other ions were interpreted as weakening the hydrogen bond, and it was found that the effect of anions on the hydrogen bond was greater than that of cations. Wang et al.^14^ divided these ions into two categories according to their influence on water structure. One is to association the hydrogen bonds water molecules in the hydration layer; the other is to destroy the hydrogen bonds between water molecules in the hydration layer, and the effect is related to viscosity coefficient *B*_*η*_:

$$\eta /{\eta }_{0}=1+A{c}^\frac{1}{2}+{B}_{\eta }c+D{c}^{2}$$

where *η* and *η*_*0*_ are the viscosities of electrolyte solution and pure water, respectively, and *B*_*η*_ is the electrolyte’s viscosity coefficient. The solution’s characteristic constants are *A* and *D*. The concentration of the solution denoted by *c*. *A* value can be used to characterize the interaction of solutes in a solution, which can be ignored in this study. The *D* value is related to solute-to-solute and solute-to-solvent interactions in solution and can be ignored in this case. As a result, the electrolyte’s viscosity coefficient can be calculated by measuring the viscosity of the solution and pure water. Typically, the electrolyte’s viscosity coefficient that weakens hydrogen bonds between water molecules is less than zero, while the electrolyte’s viscosity coefficient that strengthens hydrogen bonds between water molecules is greater than zero.

In this study, the influence of anions on hydrogen bonds of water molecules in the hydration layer was explored in the range of THz, and four common ammonium salts solutions (0.9 mol/L NH_4_Cl, (NH_4_)_2_SO_4_, (NH_4_)_2_CO_3_, CH_3_COONH_4_) at the same concentration were selected, and because the cations in the solution are the same, anions play an important role in the change of hydrogen bond between water molecules. The THz frequency domain spectrum information of the solutions obtained by multiple measurements, the order of the spectrum intensity is: CH_3_COONH_4_ > (NH_4_) _2_SO_4_ > (NH_4_) _2_CO_3_ > NH_4_Cl. Given that the influence of water on THz absorption is primarily due to the hydrogen bond between water molecules, and it is found that the spectral intensity of the four ammonium salts is lower than that of distilled water. Therefore, it can be inferred that the four ammonium salt solutions promote the formation of hydrogen bonds between water molecules, thereby reducing the THz spectrum’s intensity. Based on the above theory, the viscosity coefficients *B*_*η*_ of SO_4_^2−^ and CO_3_^2−^ are calculated to be 0.206 and 0.294, respectively, indicating that both anions promote the formation of hydrogen bonds between water molecules, and CO_3_^2−^ is larger than SO_4_^2−^, which is consistent with THz spectroscopy experimental results. It can be inferred that CH_3_COO^−^ and Cl^−^ can also form hydrogen bonds between water molecules, and the order of the association effect of four anions on the molecular hydrogen bond in the hydration layer from strong to weak is Cl^−^ > CO_3_^2−^ > SO_4_^2−^ > CH_3_COO^−^.

### Effect of external electric field on THz transmission characteristics of ammonium salt solution

It is well known that the strong absorption of THz waves by water comes mainly from the resonant absorption of hydrogen bonds around water molecules, which largely limits the application of THz technology in the field of biochemistry. However, the distribution of hydrogen bonds in water is dynamic in nature and is susceptible to the influence of external factors. As mentioned above, with the introduction of ammonium salts, ionic hydration occurs in solution and the super-strong electric field of approximately 10^8^ V/cm^14^ near the ion changes the structure of the water. Inspired by this phenomenon, we carried out the effect of an applied electric field on the THz transmission properties of ammonium salt solutions.

**Figure 11:**
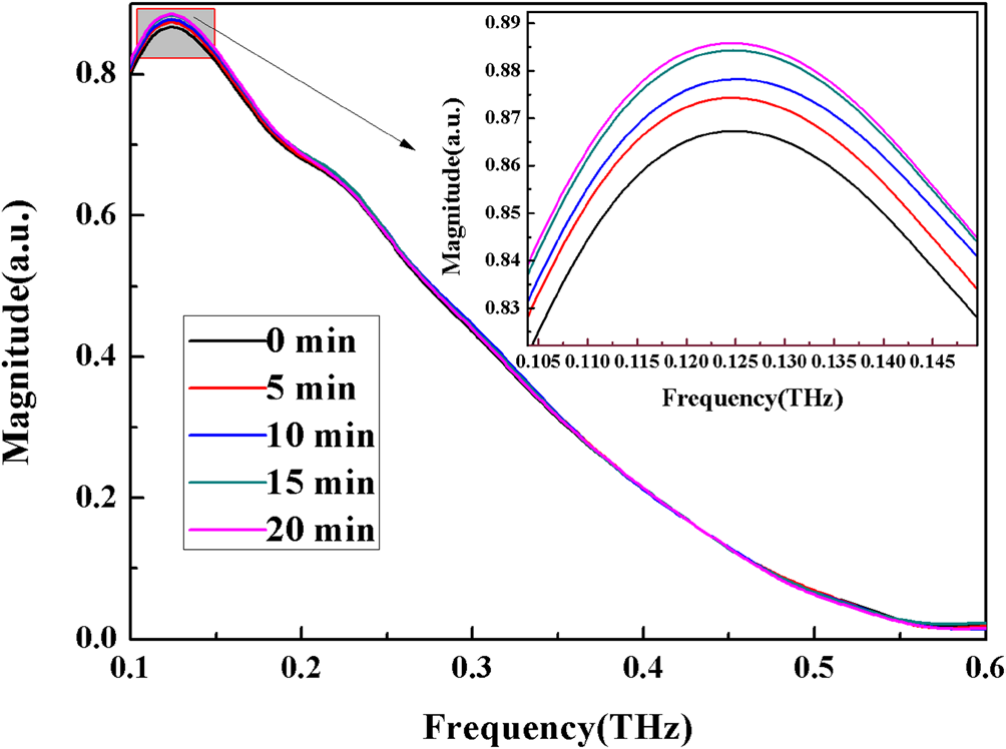
Frequency domain spectrum of H_2_O solution with different time electric field applied externally.

Zong et al.^15^ used MD simulations under a very high external electric field of 9 × 10^6^ V/cm to probe the change in the viscosity component of water and the anisotropy due to redistribution of hydrogen bonds in water. On this basis, we used the chip to study the THz characteristics of water under the applied electric field at different times. The experimental results are shown in Fig. 11.

The external electric field strength in this experiment is only 2500 V/cm, which is much lower than the simulated value, so no significant change in the viscosity of the water and the four ammonium salt solutions was found, nor was anisotropy caused by redistribution of hydrogen bonds. However, we can see from Fig. 11 that the THz transmission intensity of H_2_O gradually increases with the increase of the applied electric field time and reaches saturation at about 15 min. This is due to the fact that water as a polar molecule has electric dipole interactions. When an electric field is applied, the dipole moment rotates at different angles which in turn affects the vibration, rotation and spatial structure of the entire water molecule, causing changes in THz transmission. Under the action of uniform electric field, the electric dipole of water molecules will be affected by the moment, and the relationship between them is:

$$M={\mu }_{0}ESin\theta $$

where *M* is the torque of the water molecule under the action of the applied electric field; μ_0_ is the natural dipole moment of water molecule. *θ* is the angle between the electric field intensity *E* and the natural dipole moment *μ*_*0*_.

With the introduction of the ammonium salts, ionic hydration occurs first in the solution, forming an ionic hydration layer and changing the structure of the water. After the application of the electric field, the molecular orientation of the four ammonium salts undergoes a reorientation, and with the increase in time of the applied electric field, the orientation gradually increases, the solution dielectric constant decreases, and the peak of the O-H radial distribution function in the electrolyte solution has a slightly lower peak compared to the case without the applied electric field^16^. It is shown that the electric field weakens the hydrogen bonding in the ammonium salt solution. The structure of the original clusters in the water changes as well. The large clusters gradually dissociate into smaller clusters of water molecules, the number of individual water molecules increases, their activity increases and the diffusion coefficient of water molecules increases, which hinders ionic bonding in solution. Thus, due to the applied electric field, the orientation of the four ammonium salt molecules became larger and the THz transmission intensity of the solution increased with time and did not show a saturation phenomenon similar to that of water, which we attribute to the introduction of solute molecules.

## Discussion

In this study, an embedded COC THz microfluidic chip with automatic sampling function is designed. The chip is simple to prepare and easy to operate. The THz spectra of distilled water and NH_4_Cl, (NH_4_)_2_SO_4_, (NH_4_)_2_CO_3_, CH_3_COONH_4_ are obtained in the 0.1–0.6 THz range, demonstrating the chip’s practicability.

The experimental results revealed that, when the selected salt solution cations are ammonium ions, the order of spectral intensity is: CH_3_COONH_4_ > (NH_4_)_2_SO_4_ > (NH_4_)_2_CO_3_ > NH_4_Cl, and all are lower than the spectral intensity of distilled water, indicating that all four anions play a role in promoting hydrogen bonding between water molecules. The THz transmission intensity of the four ammonium salt solutions increased gradually with the increase of the applied electric field time (the absorption coefficient decreased gradually), indicating that the hydrogen bond in the solution would be damaged by the applied electric field and the destruction effect would increase with time. This study lays the groundwork for future THz spectroscopy applications in biochemistry.

## Supplementary Information

Supplementary Figures.
