# Supplementary material for: Terahertz absorption characteristics of ammonium salt solution based on self-sampling microfluidic chip
Source: Sci Rep. 2022 May 17;12:8144. doi: 10.1038/s41598-022-11858-6 (PMC9114126; doi:10.1038/s41598-022-11858-6)
Supplement: Supplementary file 1 — Supplementary Figures. [file 41598_2022_11858_MOESM1_ESM.docx]

Supplementary material


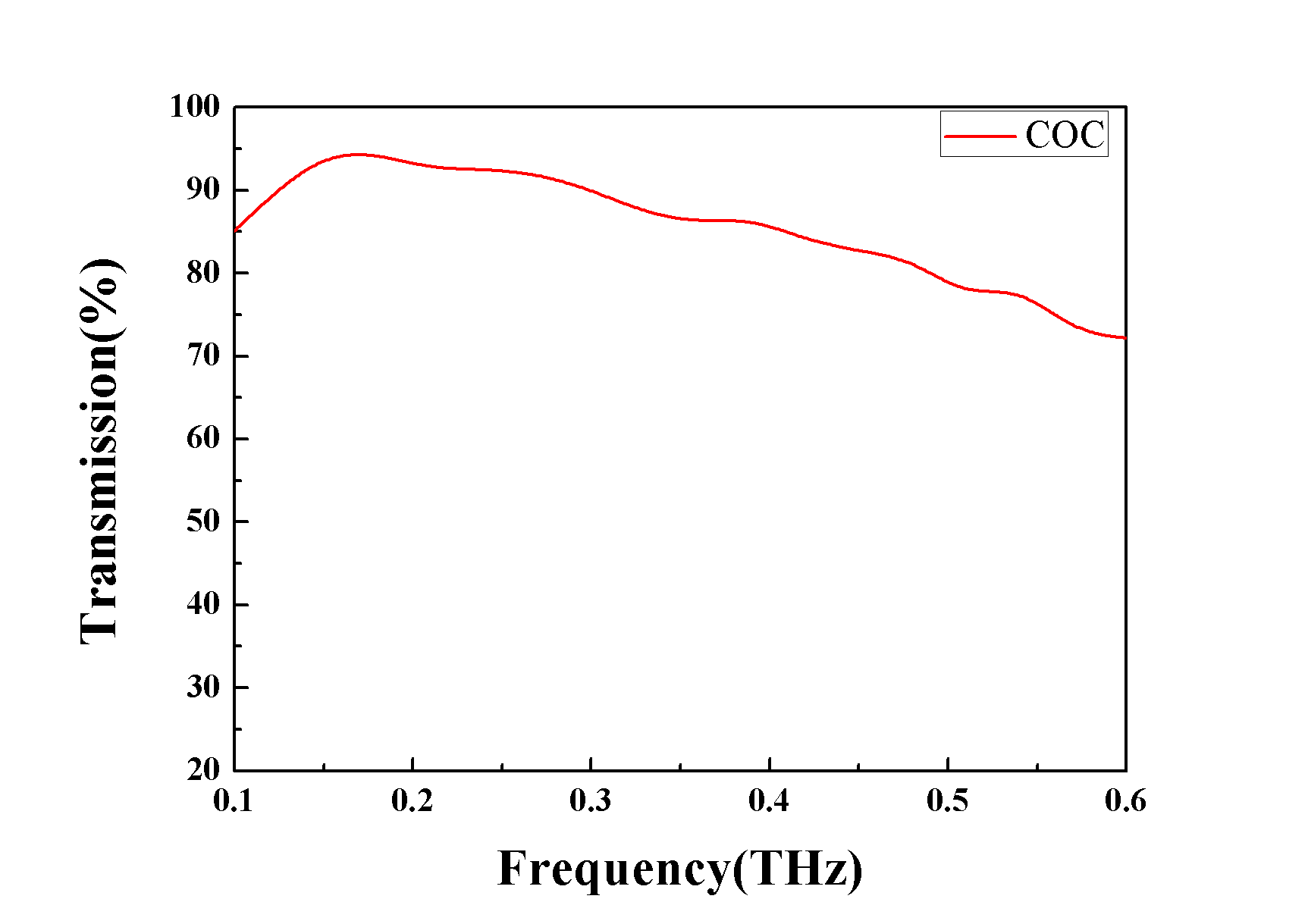


**Figure S1.** THz transmission spectrum of COC material


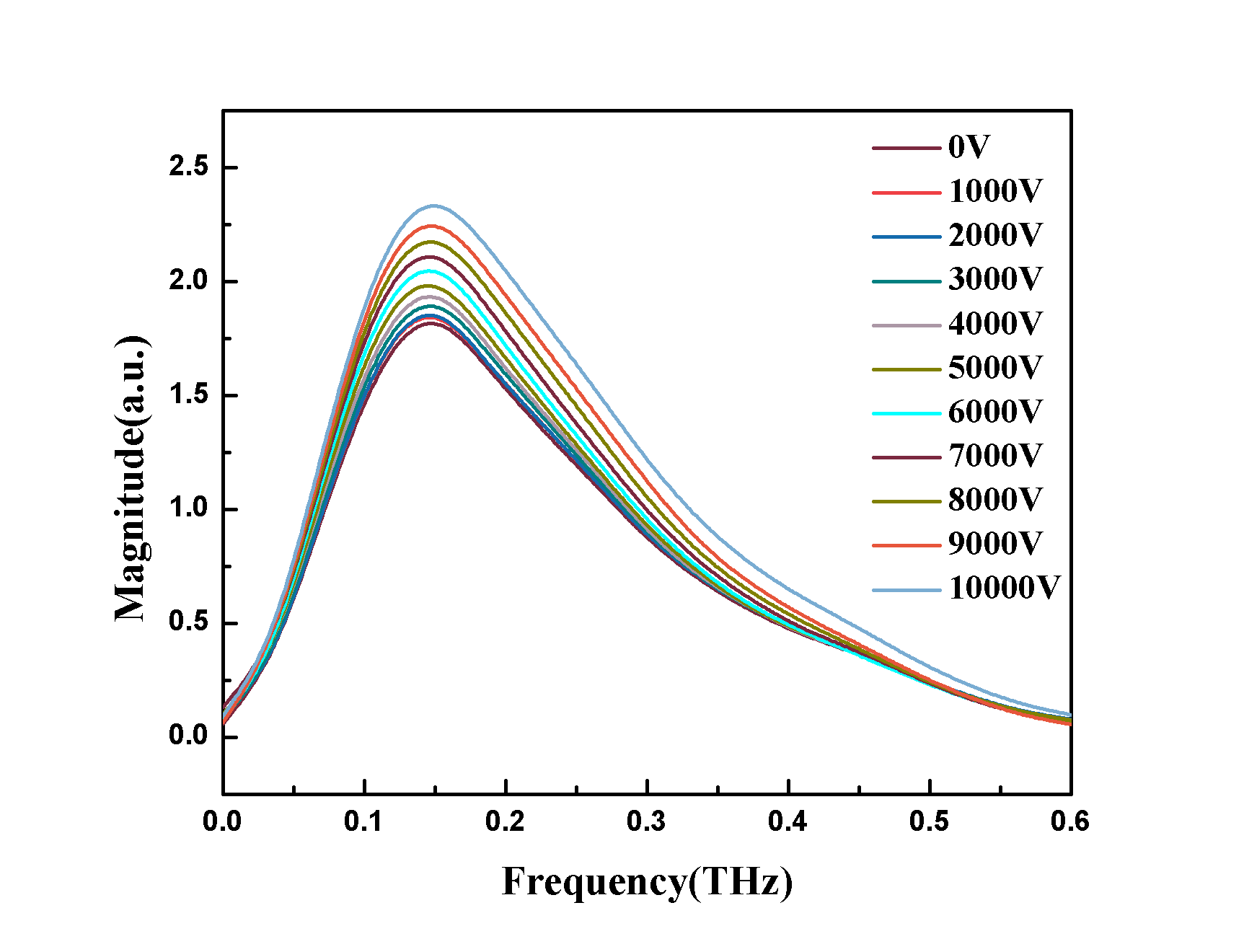


**Figure S2.** THz frequency domain spectrum of C_6_H_5_Li_3_O_7_ solution with different strengths electric field applied externally


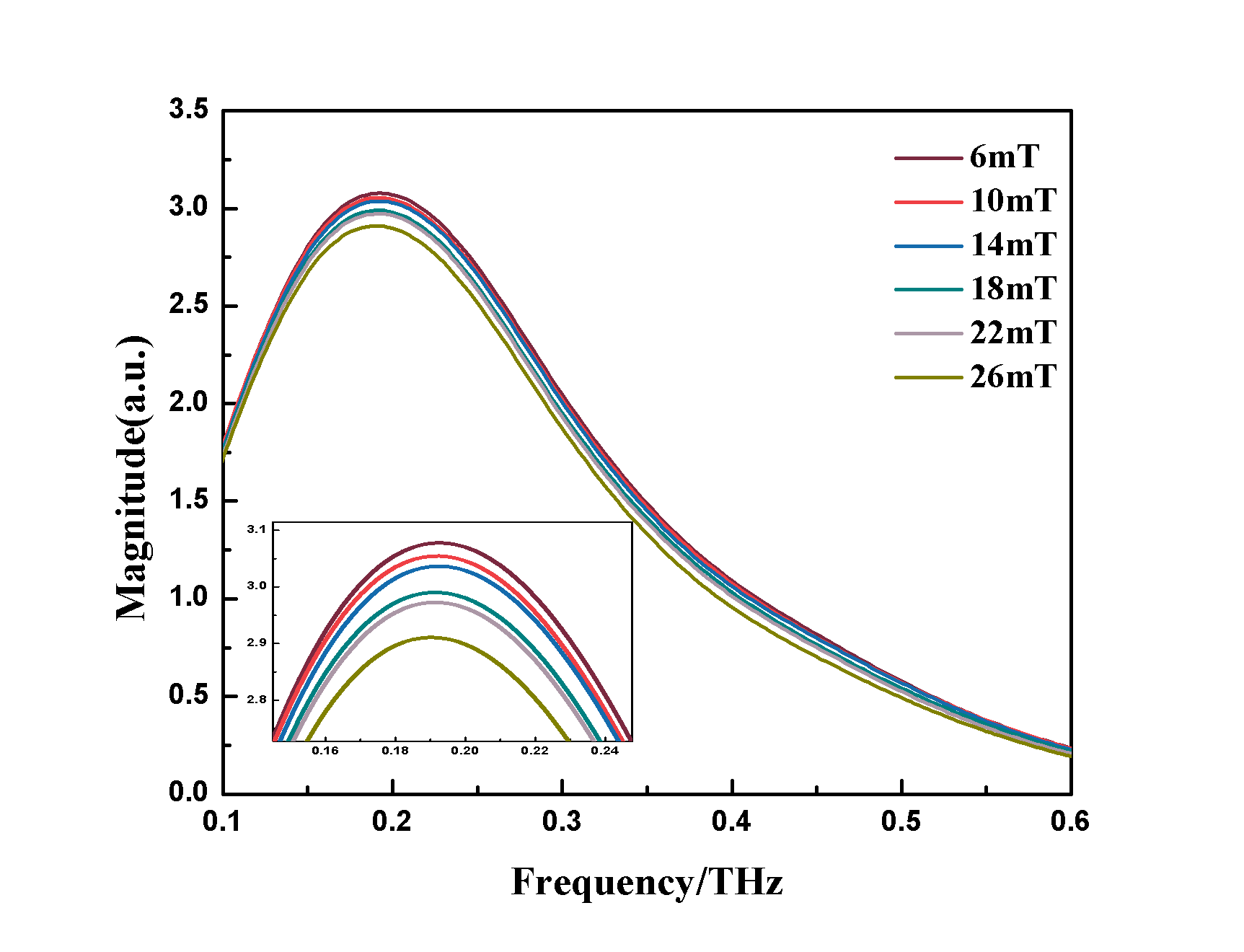


**Figure S3.** THz frequency domain spectrum of C_6_H_5_Li_3_O_7_ solution with different strengths magnetic field applied externally

During the experiment, we increased the electric and magnetic field in larger steps, and it can be seen from the figure that the frequency domain spectrum amplitude increased uniformly with increasing electric and magnetic field strength over the range of field strengths we have studied, and no obvious threshold appeared.
